# PEG Induces High Expression of the Cell Cycle Checkpoint Gene *WEE1* in Embryogenic Callus of *Medicago truncatula*: Potential Link between Cell Cycle Checkpoint Regulation and Osmotic Stress

**DOI:** 10.3389/fpls.2017.01479

**Published:** 2017-09-05

**Authors:** Adel M. Elmaghrabi, Hilary J. Rogers, Dennis Francis, Sergio J. Ochatt

**Affiliations:** ^1^Biotechnology Research Center Tripoli, Libya; ^2^School of Biosciences, Cardiff University Cardiff, United Kingdom; ^3^Agroécologie, AgroSup Dijon, Institut National de la Recherche Agronomique (INRA), University of Bourgogne Franche-Comté Dijon, France

**Keywords:** cell cycle, cell division, cell morphology, gene expression, legumes, *Medicago truncatula*, water stress, *WEE1*

## Abstract

Polyethylene glycol (PEG) can be used to mimic osmotic stress in plant tissue cultures to study mechanisms of tolerance. The aim of this experiment was to investigate the effects of PEG (M.W. 6000) on embryogenic callus of *Medicago truncatula.* Leaf explants were cultured on MS medium with 2 mg L^-1^ NAA and 0.5 mg L^-1^ BAP for 5 months. Then, calli were transferred to the same medium further supplemented with 10% (w/v) 6000 PEG for 6 months in order to study physiological and putative molecular markers of water stress. There were no significant differences in growth rate of callus or mitotic index ± PEG although embryogenic potential of PEG treated callus was morphologically enhanced. Cells were rounder on PEG medium and cell size, nuclear size and endoreduplication increased in response to the PEG treatment. Significant increases in soluble sugar and proline accumulation occurred under PEG treatment compared with the control. Significantly, high *MtWEE1* and *MtCCS52* expression resulted from 6 months of PEG treatment with no significant differences in *MtSERK1* or *MtP5CS* expression but down regulation of *MtSO*S expression. The results are consistent in showing elevated expression of a cell cycle checkpoint gene, WEE1. It is likely that the cell cycle checkpoint surveillance machinery, that would include *WEE1* expression, is ameliorating the effects of the stress imposed by PEG.

## Introduction

Water stress can result in reducing crop yield world-wide (Boyer, 1982; Smirnoff, 1993; Gonzalez et al., 1995) and a recent UN survey has underlined the importance of water deficit in our planet and its effects for the coming generations unless urgent measures are taken. This situation is exacerbated in arid and semiarid ecosystems. Here, legumes play a central agroecological role through their ability to use atmospheric nitrogen via the symbiosis with Rhizobia, and thus it reduces the need for fertilizers, improve food security, and generally favor the environment (Rubio et al., 2002; Naya et al., 2007; Kohler et al., 2008; Araújo et al., 2015; Ochatt, 2015). Studying a legume model species is thus timely and *Medicago truncatula* is of particular interest given its rather short life cycle and autogamy. It has a small and almost completely annotated genome (500–550 Mbp) which is publicly available (Goodstein et al., 2012), and it is more drought tolerant than other legume crops such as pea, bean, and soybean (Motan et al., 1994; Gonzalez et al., 1998; Costa França et al., 2000; Galvez et al., 2005). In spite of this, previous studies on water stress resistance in *M. truncatula* mostly concerned gene transfer (Alcântara et al., 2015; Araújo et al., 2015; Duque et al., 2016). The assessment of physiological responses (Nunes et al., 2008) and their genetic mechanisms (Badri et al., 2011) is more limited.

Osmotic stress or water deficit can be defined as the absence of adequate moisture necessary for a plant to grow normally and complete its life cycle (Cabuslay et al., 2002). Resistance mechanisms can be grouped into three categories: firstly escape, which enables the plant to complete its life cycle before the most intense period of water shortage, secondly avoidance, which prevents exposure to water stress, and finally tolerance, which enables the plant to withstand stress conditions (Levitt, 1972; Zhu, 2002; Golldack et al., 2014). Some resistance mechanisms are constitutive and active before exposure to water shortage. In other cases, plants exposed to water stress alter their physiology, thereby acclimating themselves to withstand drier conditions. One of the tolerance mechanisms activated under such stress is that of mitigating osmotic stress, via the production of osmolytes such as proline, and soluble sugars, that protect cells against osmotic perturbation (Choudhary et al., 2005; Valliyodan and Nguyen, 2006; Fulda et al., 2011; Elmaghrabi et al., 2013; Deinlein et al., 2014). On the other hand, φw (water potential) is also known to induce a morphological variation in tissues subjected to osmotic stress, notably at the cellular level. Such variation is potentially useful to understand biodiversity by identifying cellular responses to stress that are not necessarily picked up by taxonomic or phylogenetic indices that consider cell shape or size *in vitro* (Ochatt et al., 2008; Ochatt and Moessner, 2010). It is also important for assessing the competence for regeneration *in vitro* (Ochatt et al., 2008) following the recovery of tissues with a novel genetic makeup obtained via *in vitro* selection (Elmaghrabi et al., 2013) or gene transfer (Alcântara et al., 2015).

Responses to abiotic stress factors involve a reprogramming of the expression of 1000s of genes, which in turn result in the modification of a range of cellular and physiological processes (Cushman and Bohnert, 2000; Sreenivasulu et al., 2004; Araújo et al., 2015). One example of tolerance to stress at the molecular level, is the induction of *P5CS* that encodes Δ^1^-pyrroline -5-carboxylate synthetase involved in proline biosynthesis (Silva-Ortega et al., 2008). This gene is highly expressed in salt-and drought-tolerant plant species (Choudhary et al., 2005) and it is induced under salt and water stress in many plant species including legumes (Chen et al., 2009). The *P5CS* gene was also up-regulated in *M. truncatula* in response to salt stress (Elmaghrabi et al., 2013). The kinetics of expression of genes involved in the cell cycle in plants exposed to high levels of abiotic stress has been the object of a number of studies (Gill and Tuteja, 2010; Zhao et al., 2014; Roy, 2016). In Arabidopsis, a negative regulator of mitosis, *WEE1*, is strongly expressed in response to abiotic stress (De Schutter et al., 2007). Osmotic stress imposed using polyethylene glycol (PEG) also up-regulated oxidative DNA damage and consequently DNA repair enzymes both in imbibed seeds (Balestrazzi et al., 2011) and in plantlets (Macovei et al., 2010). Our recent work with *M. truncatula* also showed an increased expression of *WEE1* and *CCS52* (*CELL CYCLE SWITCH PROTEIN 52*, another gene involved in the cell cycle) in salt-acclimated tissues as well as expression of genes involved in salt tolerance (*SOS1* encoding a Na^+^/H^+^ antiporter) and embryogenesis *in vitro* [*SOMATIC EMBRYOGENESIS RECEPTOR-LIKE KINASE 1* (*SERK1*)] (Elmaghrabi et al., 2013).

Tissue culture has been used in the selection of water stress tolerant cell lines that have been used to regenerate plants resistant to harsh environmental conditions in a range of crops including *Medicago sativa* L., tomato, soybean, and wheat (Sakthivelu et al., 2008; Guóth et al., 2010; Mahmood et al., 2012). Water deficit *in vitro* can be imposed through treatment with PEG 6000 (Ochatt et al., 1998; Guóth et al., 2010; Rai et al., 2011; Yang et al., 2012). The adsorbant property of this inert osmolyte provokes in plant cells and tissues the same or comparable effects to those obtained by drying soil at the same φw and without any other associated detrimental effects (Michel and Kaufmann, 1973). PEG 6000 thus closely mimics soil water stress (Lu and Neumann, 1998) and induces increases in total soluble sugars which serve as an osmoticum, or can be a source of respiratory substrates (Srivastava et al., 1995; Elmaghrabi et al., 2013). Additionally, PEG was shown to stimulate somatic embryogenesis *in vitro* (Attree et al., 1995; Igasaki et al., 2003). PEG 6000 was also used, and at similar concentrations as here (although osmolarity was expressed in MPa rather than in mOsm/kg as in this work), in studies on PEG-induced DNA damage with *M. truncatula in vitro* plantlets (Macovei et al., 2010) and seeds (Balestrazzi et al., 2011).

As many legumes are grown (or have their center of origin) in regions with an arid to semi-arid climate (Smýkal et al., 2015), a number of studies have identified genes, QTLs, ESTs, and SNPs that are responsive to drought stress in several species (Jacob et al., 2016). However, the molecular basis of water stress tolerance is not fully understood in *M. truncatula* (Araújo et al., 2015). The aim of the current work was to examine the extent to which treatments with PEG could enhance osmotic stress tolerance potential in callus of *M. truncatula*. In addition, the accumulation of osmoprotectants, the effects on cell morphology (shape and size) and division competence, and the expression of *WEE1, CCSS52, P5CS*, and *SOS1* were monitored in the PEG treatments to investigate the mechanism underlying the induced water stress responses as compared to those activated in response to salt stress (Elmaghrabi et al., 2013). The expression of *SERK1* was also analyzed, given its key role on the competence for the subsequent regeneration through somatic embryogenesis of plants that may potentially carry the stress resistance trait acquired.

## Materials and Methods

### Plant Material

*Medicago truncatula* cv. Jemalong line A17 (2n = 2x = 16, 1C value = 0.48 pg) was used in this study. One hundred leaves were explanted to tissue culture from 4 week old aseptically grown plants onto a medium consisting of MS basal medium (Murashige and Skoog, 1962) with 2 mg L^-1^ NAA (alpha-naphthalene acetic acid; Sigma, Poole, United Kingdom) and 0.5 mg L^-1^ BAP (6-benzylaminopurine; Sigma, Poole, United Kingdom), hereafter called MANA medium as in Elmaghrabi et al. (2013), and dispensed in multi-well dishes as 2 mL aliquots per well. Cultures were incubated at 24/22°C (day/night), with a 16/8 h (light/dark) photoperiod of 90 μmol m^-2^ s^-1^ from warm white fluorescent tubes. After 4 weeks, explants were sub-cultured on the medium above and the frequency of callus initiation assessed.

Leaf-derived embryogenic callus was obtained after culture on MANA medium for 5 months. Calli were screened for embryogenesis (i.e., somatic embryos at different developmental stages, identified as spherical glistening nodules when globular, through to elongated greening structures at later stages) or, organogenesis (development of shoots and/or roots), as reported elsewhere (Ochatt et al., 1998, 2013; Elmaghrabi and Ochatt, 2006; Ochatt and Revilla, 2016). Only embryogenic calli were transferred onto 25 ml of MANA medium supplemented with or without 10% w/v (-0.66 MPa) PEG6000 (PEG; Sigma, Poole, United Kingdom) for 6 months in order to acclimate the cultures under conditions that mimic water (osmotic) stress (at least 12 calli per treatment). This PEG concentration was chosen based on previous studies with various species (Biswas et al., 2002; Guóth et al., 2010) and also including *M. truncatula* (Macovei et al., 2010; Balestrazzi et al., 2011). Growth data (g fresh weight, g FW) were recorded and results were statistically analyzed (*P* ≤ 0.05; Kruskal–Wallis followed by a Dunn’s test).

### Proline and Water Soluble Carbohydrate Measurements

Proline content was measured as described in Elmaghrabi et al. (2013) and according to Troll and Lindsley (1955) and Boukel and Houassine (1997) from callus tissue (100 mg per sample per treatment) grown on 0 and 10% (w/v) PEG. All treatments were repeated three times. Optical density was measured using a spectrophotometer (UNICAM; Cambridge, United Kingdom) at a wavelength of 528 nm and calibrated using a standard curve of proline solutions (0.1–0.4 mg mL^-1^; Sigma, Poole, United Kingdom).

Determination of soluble sugars was by the anthrone method (Plummer, 1987; Elmaghrabi et al., 2013) using 100 mg callus samples from 0 and 10% (w/v) PEG treatments (three replicates). The soluble sugar content was measured spectrophotometrically (UNICAM, Cambridge, United Kingdom) at 585 nm and the data were converted to mg L^-1^ using the calibrations established prior to the assay.

### Medium and Callus Osmolarity

For measurements of medium and callus osmolarity a Wescor (model VAPRO 5520, South Logan, UT, United States) vapor pressure micro-osmometer was used and a minimum of three 10 μL samples were measured. For medium osmolarity assessments, 10 mL of the medium were vortexed prior to collecting the 10 μL samples to be measured. For callus osmolarity measurements, 1 g fresh weight of tissue was collected in 2 mL of liquid medium and centrifuged (100 g, 10 min, 10°C). The supernatant was carefully removed, the pellet was crushed in an Eppendorf with a pestle and centrifuged (1000 *g*, 10 min, 4°C), and this second supernatant was finally employed for measurements of osmolarity. Results from such measurements, expressed in mMkg^-1^, are the mean + SE of a minimum of three individual samples per treatment, and were performed at the time of sub culturing and over at least three consecutive subcultures.

### Mitotic Index, Cell Viability, and Cell Morphology

For determinations of C-value stability of calli following *in vitro* selection for several months they were compared to leaf tissues from the original plants. Nuclei were mechanically isolated from about 0.2 g of calli or from a single leaf of *M. truncatula* A17 grown in green house conditions. Tissues were chopped roughly with a sharp razor in 400 μl of nuclei extraction buffer and 1.6 mL of staining buffer (Partec^®^; Canterbury, United Kingdom) (Ochatt, 2008). The suspension was filtered through a 20 μm nylon mesh and 4, 6 diamidino-2-phenylindole (DAPI; Sigma, Poole, United Kingdom), an A-T binding specific fluorochrome, was added to the filtrate to a final concentration of 1 μg mL^-1^. The DNA contents of the isolated nuclei suspension were analyzed using a Partec PAS-II flow cytometer equipped with an HBO-100 W mercury lamp and a dichroic mirror (TK420). Ten replicated calli for each treatment were analyzed, with a minimum of 3000 to 10000 nuclei per run. The mitotic index was calculated according to the formula: MI = 4 x 4C/Σ 2C + 4C, where 2C and 4C correspond to the mean integrated value of nuclei in G1 phase and G2, respectively (Ochatt, 2008).

Cell viability was estimated by dual staining with fluorescein diacetate (FDA; Sigma, Poole, United Kingdom) and propidium iodide (PI; Sigma, Poole, United Kingdom). Cell suspensions (75 μL) from each treatment were mixed with 75 μL of dual staining solution containing FDA (200 μg mL^-1^; Widholm, 1972) and propidium iodide (PI at 120 μg mL^-1^) on ice and incubated for 20 min. The FDA molecule is cleaved by the esterases in the cytoplasm into acetate and fluorescein which, being hydrophilic accumulates in the cytoplasm of metabolically active (alive) cells that, upon excitation with the UV light fluoresce yellow–green, while dead cells appear red using a fluorescent microscope. A minimum of 300 cells are counted and results are expressed as the percentage of fluorescing cells referred to the total number of cells in the field.

For the cell morphology characterization, FDA stained slides of the control and PEG-treated cells were observed under the microscope under the UV. The surface area of cells and nuclei was determined at 2, 4, and 6 months of culture, using the image acquisition programs ArchimedPlus and Histolab (Microvision, France) as reported (Ochatt et al., 2008), and a shape coefficient (Ochatt and Moessner, 2010) was applied at 6 months of culture. Briefly, this shape coefficient (SC) is calculated based on the half length of the cell along its longest (a) and shortest (b) axes, as:

$$\text{SC=}\frac{\sqrt{{\text{a}}^{2}-{\text{b}}^{2}}}{\text{a}}$$

For each treatment, nucleus and cell size were measured on 10 cells at 2 and 4 months of culture and at least 20 cells at 6 months of culture, and results expressed as the mean ± SE.

The SC distinguishes round from elongated shapes, since SC values close to 1.0 correspond to elongated cells while SC values close to 0.5 correspond to rounder cell shapes.

### Real Time PCR

RNA was extracted and genomic DNA removed by DNase treatment (Spadafora et al., 2012; Elmaghrabi et al., 2013), and its absence verified using 18S rRNA primers (Spadafora et al., 2011). Retrotranscription was carried out using an Ambion kit (RETROscript Reverse transcription for RT-PCR; Foster City, CA, United States) and 2 μg of RNA. An ABsoluteTM QPCR SYBR Green Mix (Thermo Scientific, Waltham, MA, United States) kit was used for real time PCR. Reactions (in a total volume of 25 μL) consisted of: 5 μL cDNA (1:20 dilution), 12.5 μL ABsoluteTM QPCR SYBR Green Mix, 1.75 μL of each primer (10 μM) and 4 μL H_2_O. Reactions were cycled in an MJ Research OPTICON 2 (Quebec, Canada), in triplicate under the following conditions: 95°C for 10 min, 40 cycles of: 95°C for 15 s, 60°C for 30 s and 72°C for 30 s and one cycle of 72°C for 30 s. For testing primer specificity, a melting curve analysis was performed after amplification (from 60 to 98°C with an increasing heat rate of 0.5°C s^-1^). A relative quantification of gene expression was calculated using the 2-DDCT method (Livak and Schmittgen, 2001). Primers for the target genes: *MtSOS1, MtWEE1, MtSERK1, MtP5CS*, and *MtCCS52* are as described in Elmaghrabi et al. (2013). Mt18S rRNA primers were used to normalize the results as it was shown previously that 18S rRNA was a reliable reference gene for stress responses in *M. truncatula* (Elmaghrabi et al., 2013), and widely used across a range of different species for developmental and stress-response studies (e.g., Price et al., 2008; Wagstaff et al., 2010).

### Statistical Analyses

Unless otherwise stated above, data were analyzed using R software (R version 3.3.2, Foundation for Statistical Computing). One or two way (as appropriate) ANOVA tests followed by a Tukey’s test, or non-parametric statistical tests (Kruskal–Wallis followed by a Dunn’s test) were applied to determine differences across multiple samples. Comparisons between pairs of samples were performed using a Student’s *t*-test, or if not normally distributed using a Wilcoxon signed rank test. Regression equation and *R*^2^ value for the growth data were calculated in Excel. Details of tests applied are provided in the legend to each Figure and all original data are provided as Supplementary Files S1–S6.

## Results

### PEG Enhanced Callus Phenotype and Embryogenic Competence without Reducing Its Viability

**FIGURE 1:**
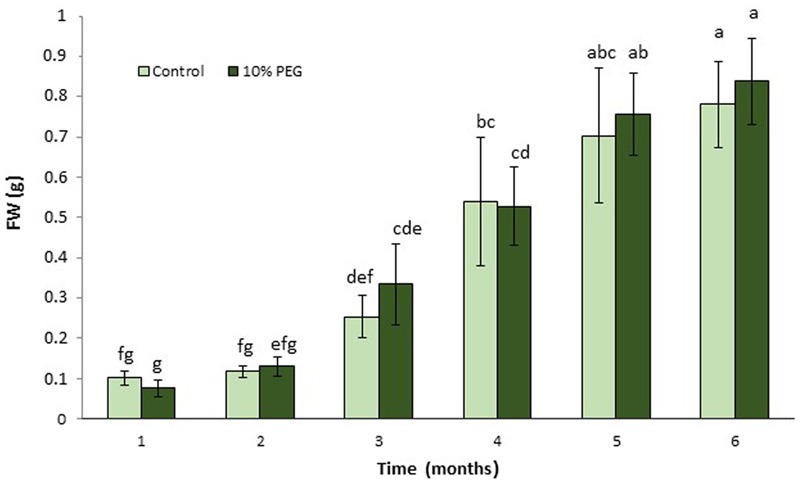
Mean (± SD) callus growth over 6 months on MANA medium with 10% w/v PEG compared with control (MANA medium). Different letter combinations indicate significant differences (*P* ≤ 0.05; Kruskal–Wallis followed by a Dunn’s test) (*n* > 8). See Supplementary File S1 for data and statistical analyses.

**FIGURE 2:**
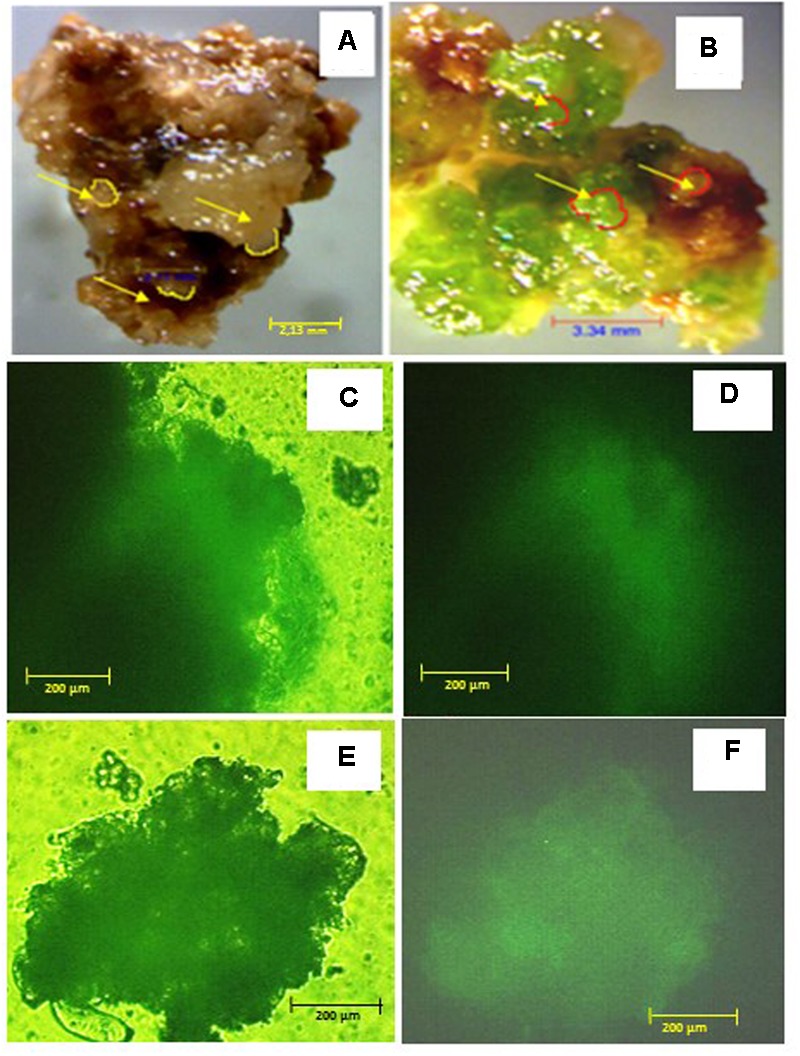
Callus phenotype and viability after 6 months on 10% w/v PEG and control media: **(A)** control callus; **(B)** PEG-selected callus, arrows indicate somatic embryos; viability of control callus after FDA staining observed under transmission **(C)** or UV **(D)** light; viability of PEG-selected callus after FDA staining observed under transmission **(E)** or UV **(F)** light. Scale bars are **A** = 2.13 mm, **B** = 3.34 mm; and **C–F** = 200 μm.

There were no significant differences between *M. truncatula* leaf callus cultured on MANA medium or MANA medium supplemented with 10% w/v PEG6000 over the 6 months of culture, and the linear growth rates for control and PEG treatments were 0.155 and 0.168 g month^-1^ respectively (**Figure 1**). This only represents a 1.08 fold increase in the PEG compared with the control treatment indicating very similar rates of growth in each treatment irrespectively of the presence or absence of PEG in the culture medium. However, these data also suggest that callus tissues were PEG-tolerant already within 1 month of sub-culture and retained such tolerance throughout the experiment. Qualitative observations of callus indicated that those treated with PEG were typically bright green in color and exhibited clear evidence of embryogenesis as did also the controls; however, the controls were brown in color and the somatic embryos regenerated looked blocked at an early (globular to heart) developmental stage (**Figures 2A,B**). The typical bright green coloration of calli in the PEG treatment would tend to indicate their robustness for both growth and embryogenesis regardless of the length of time in culture, and could also be ascribed to an increased tissue photochemistry linked to the tolerance acquired by onset of a priming process by the long-term culture on PEG. This was confirmed during the cell viability assessments with fluorescein diacetate where calli grown on 10% w/v PEG for 6 months contained 81.00 + 1.5% viable cells compared to 68.00 + 5.2 for calli grown only on the MANA medium (**Figures 2C,D**).

### Mitotic Index, Cell and Nuclear Size Increase, and Cell Shape Changes in Calli Cultured on PEG-Medium

**FIGURE 3:**
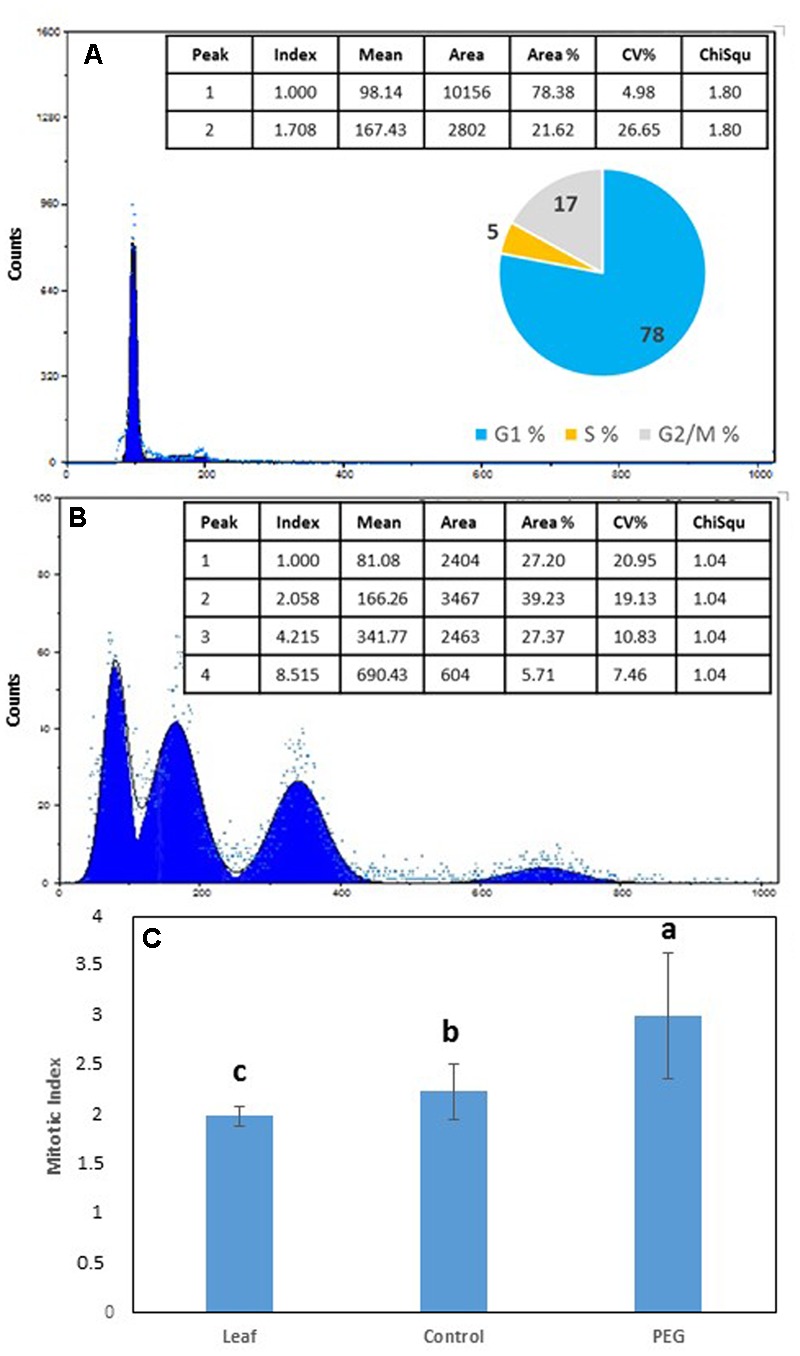
Flow cytometry (FCM) profile and % distribution of nuclei in G1 (1st peak), G2 (2nd peak), S phase (trough between 1st and 2nd peaks) and polyploid nuclei [3rd (8C) and 4th (16C) peaks] from **(A)** leaves of *Medicago truncatula*, **(B)** *M. truncatula* callus cultured on 10% w/v PEG6000 for 6 months; **(C)** Mean (± SD) mitotic index in leaves compared to callus tissues after 9 continuous months growth on 10% w/v PEG. Different letters indicate significant differences (*P* ≤ 0.05); *n* = 10. See Supplementary File S2 for data and statistical analyses.

Flow cytometry (FCM) was used to compare the C value distribution of cells from greenhouse grown *M. truncatula* leaves and callus derived from leaf material cultured for 6 months on 10% w/v PEG6000 (**Figure 3**). Flow cytometry raw profiles of leaves exhibited two peaks (**Figure 3A**), corresponding respectively to the nuclei in G1 phase (2C DNA) and those in G2/M (4C DNA), where their analysis after fitting them to Gauss curves resulted in a distribution of nuclei into three subpopulations as follows: G1 77.39%, S 17.32% and G2/M 5.29%, and coupled with a calculated mitotic index of 1.999 (**Figure 3C**). A very similar profile was obtained from calli cultured on MANA medium alone (not shown) which showed no obvious deviation from the mother plant tissues from which they originated, while the flow cytometry profiles of calli cultured on PEG was very different (**Figure 3B**). In the 10% PEG6000 treatment, four peaks were typically detected consistent with 2, 4, 8, and 16 C populations (**Figure 3B**), and indicative of the occurrence of endoreduplication. The mitotic index was also significantly higher (*P* < 0.05) for the calli grown on PEG6000 containing medium (**Figure 3C**), which is also indicative of the onset of an endoreduplication phenomenon.

**FIGURE 4:**
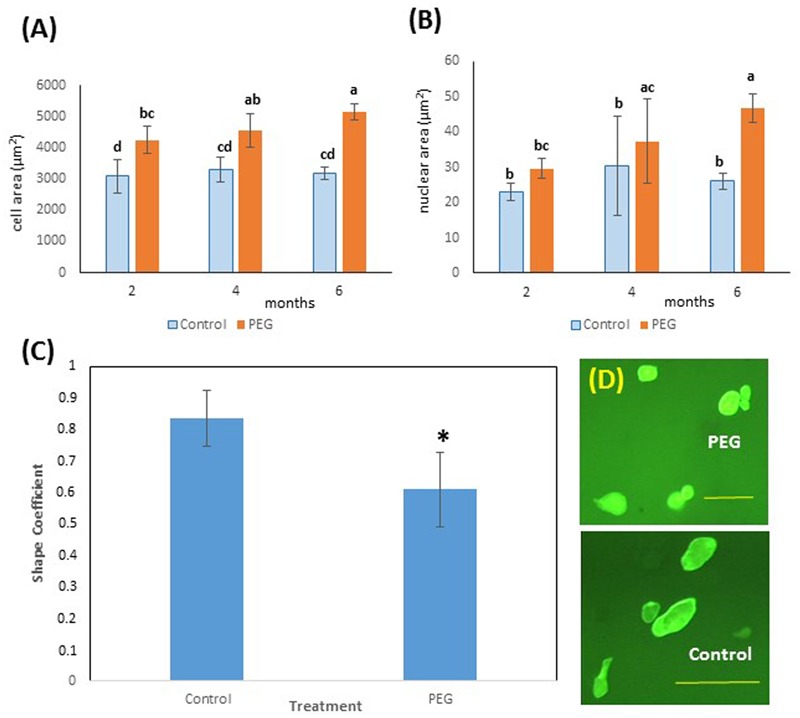
Effects of PEG6000 on cell morphology. **(A)** Cell and **(B)** nuclear size (μm^2^) at different time points during *in vitro* selection for PEG6000 (10%) resistance. Data are the means ± SD from *n* = 9 replicates at 2 and 4 months of culture and *n* = 22 measurements at 6 months of culture. Bars with different letters were significantly different (Kruskal–Wallis followed by Dunn’s test) at different time points across treatments (*P* < 0.05). See Supplementary File S3 for data and statistical analyses. **(C)** Shape coefficient (SC) of cells from control (blue) and PEG-grown (orange) calli (*n* = 22) at 6 months of mean ± SD (^∗^*P* < 0.05, Welch Two Sample *t*-test). See Supplementary File S4 for data and statistical analyses. **(D)** Images of cells from PEG and control cultures at 6 months of culture (scale bars PEG = 100 μm, Control 200 μm).

Interestingly, cell size also showed a significantly higher value for cells from calli grown on PEG6000 after just 2 months of culture (**Figure 4A**) and nuclear area was greater on PEG after 4 months (**Figure 4B**). This is consistent with the occurrence of endoreduplication, and this was coupled with a modified cell shape (**Figure 4C**), with cells grown on PEG6000 exhibiting a significantly lower SC than control cells. Thus, PEG-grown cells were consistently and significantly (*P* < 0.05) rounder (SC = 0.608 ± 0.117) than control cells which were more elongated (SC = 0.833 ± 0.090) (**Figure 4D**).

### Osmolarity, Proline and Sugar Levels Rise following PEG Treatment

**FIGURE 5:**
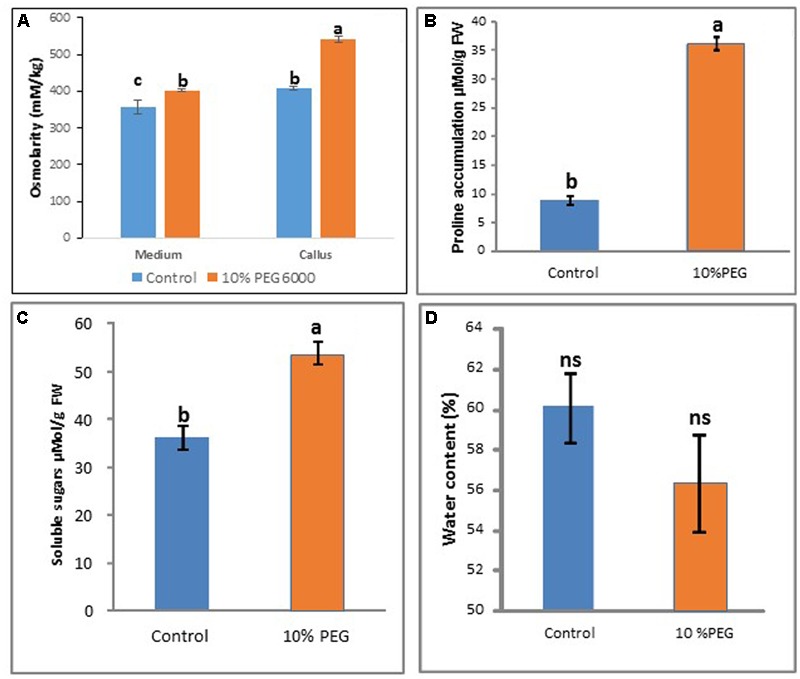
Comparisons between control and 10% PEG treatments after 6 months *in vitro* culture in terms of: **(A)** medium and callus osmolarity, **(B)** proline content, **(C)** soluble sugars, and **(D)** water content. Note different letters indicate significant differences (*P* ≤ 0.05) between treatments (*n* = 3 ± SD); ns, non-significant. [Kruskall–Wallis followed by Dunn’s test **(A)**; Wilcoxon signed rank test **(B);** and Welch Two Sample *t*-test **(C,D)**]. See Supplementary File S5 for data and statistical analyses.

After 6 months of 10% PEG 6000 treatment, there was a significant increase in osmolarity of callus in the PEG treatment compared with the control (**Figure 5A**) while osmolarity of the medium remained more constant. Proline and soluble sugar levels also increased significantly compared with the control (MANA without PEG) (**Figures 5B,C**). However there were no significant differences in water content between the PEG and control treatments (**Figure 5D**).

#### *MtWEE1* Expression Is Highly Up-regulated Following PEG Treatment While *MtSOS1* Is Down-Regulated

We chose to examine the expression of five genes as markers of processes related to osmotic (water) stress. These comprised *MtSOS1* (salt stress response), *MtWEE1* (cell cycle checkpoint), *MtSERK1* (embryogenesis) *MtP5CS* (proline metabolism), and *MtCC52* (ploidy marker) in the embryogenic calli treated with PEG. Expression of these genes was measured using quantitative real time PCR after 6 months of callus culture in PEG6000 (10%) and compared with the control treatment (0% PEG) and greenhouse grown leaves.

**FIGURE 6:**
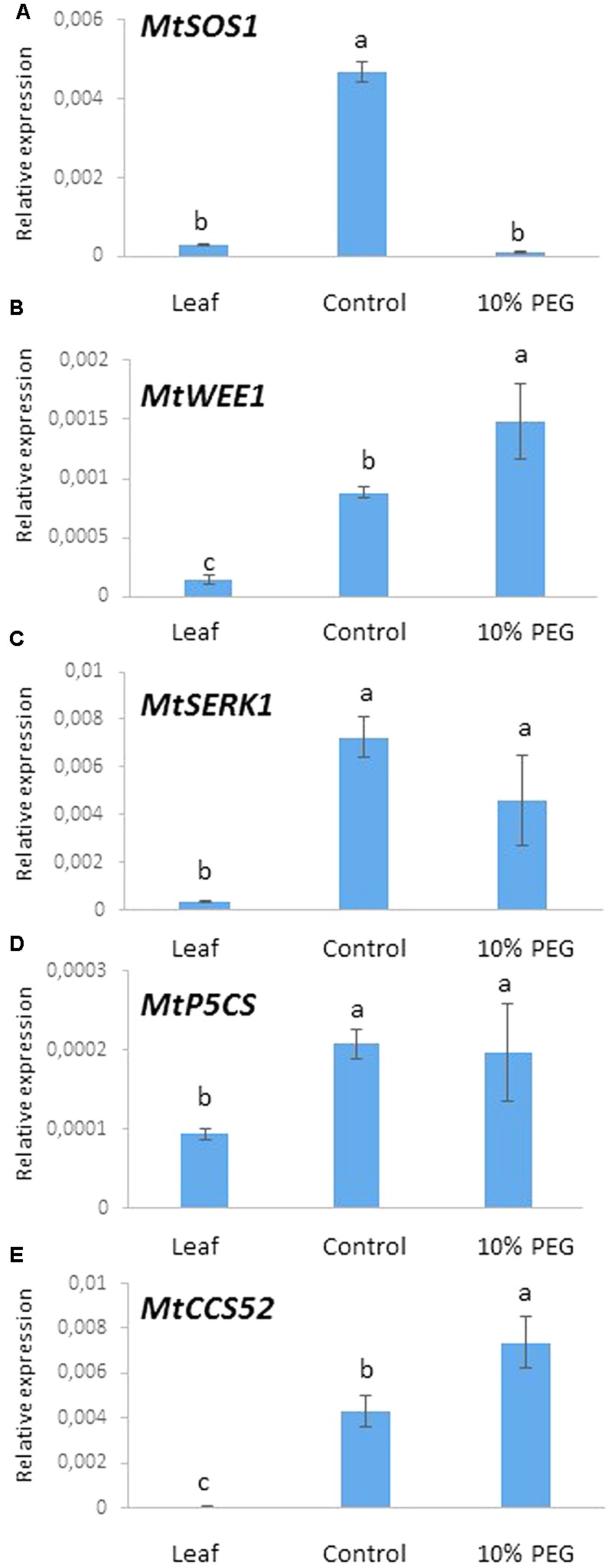
Gene expression after 6 months culture on 10% PEG medium and control (MANA medium) with leaf as reference: **(A)** *MtSOS1*, **(B)** *MtWEE1*, **(C)** *MtSERK*, **(D)** *MtP5CS*, and **(E)** *MtCCS52*. Different letters indicate significant differences amongst treatments/tissues (*P* ≤ 0.05; one-way ANOVA; *n* = 3 ± SD).

A highly significant reduction in the expression of *MtSOS1* occurred in the 10% PEG compared with the control treatment such that *MtSOS1* transcripts were virtually undetectable, comparable to expression in leaf (**Figure 6A**). Conversely, *MtWEE1* and *MtCCS52* expression was significantly higher in the PEG treated calli compared with the control treatment which in turn was higher than expression in leaf (**Figures 6B,E**). *MtSERK*, and *MtP5CS* were expressed significantly more in the control callus and 10% PEG treated callus but there was no significant difference in expression between the treated and untreated calli (**Figures 6C,D**).

## Discussion

Osmotic stress, provoked by insufficient ground and/or rain water, is a paramount constraint for plant growth and development. Cultures of callus on media that impose water deficit is a method for generating new, more tolerant, plants. Here, we have shown that long term culture of *M. truncatula* calli on medium containing 10% PEG6000 to impose an osmotic stress results in the production of morphologically enhanced calli. An analysis of protective metabolite levels, cellular morphology, cell division and gene expression was undertaken to understand the effects of the imposed stress.

In this work, callus growth was not significantly different plus or minus 10% PEG over a period of 6 months, suggesting that calli on PEG acquired tolerance to osmotic (water) stress (**Figure 1**) probably mediated by an early osmotic adjustment which was likely associated to various modifications at the cellular level (Singh et al., 2015). In fact, both cell viability and mitotic index were higher in the PEG treated cells compared to the control indicating a healthy and proliferating culture. It is likely that this sustained viability in the PEG treated cells is due to the activation of defense mechanisms that may include an activation of DNA repair as shown previously (Balestrazzi et al., 2011). The similarity in growth rates following 1 month ± PEG further stresses that tolerance was obtained relatively rapidly. This result differs from those of Biswas et al. (2002) who found that in rice, callus proliferation in the presence of PEG was greater than the controls in some genotypes, although this was at a much lower PEG concentration (5–15 g L^-1^). However in two genotypes of wheat, one drought tolerant, the other drought sensitive, water deficit decreased only slightly in the sensitive compared with the tolerant genotype under water conditions and, it did not change significantly in either the sensitive or tolerant genotype ± 400 mM PEG 6000 (100–400 mOsm; -0.976 MPa; Guóth et al., 2010). Likewise in chili pepper cultures, where there was very good growth after 12 months in 5–10% PEG8000 (0.57 MPa; Santos-Díaz and Ochoa-Alejo, 1994). Note that after 6 months of treatment, although osmolarity of the callus increased, osmolarity of the medium did not change since PEG is not metabolized. We decided to analyze both the medium and callus osmolarity as an indirect way of assessing the nutrient consumption from the medium by cells, which impacts their internal salt concentrations, as shown before with various species among which *M. truncatula* (Ochatt et al., 2008). An increased cell osmolarity appeared also to be a reliable early marker of embryogenic competence (Ochatt et al., 2008; Elmaghrabi et al., 2013). Thus the increased callus osmolarity and embryogenic capacity seen here are in line with previous observations.

In this work (**Figure 4**) PEG-induced stress resulted in a highly significant increase of the size of both nuclei and cells after 6 months of culture on selection medium. Remarkably, this was also coupled with a consistent and significant modification of the cell shape, reflected by the SC values observed, indicative of an increased elasticity of cell walls under PEG-induced osmotic stress. A similar modification of cell wall elasticity was observed in *M. truncatula* plants subjected to a severe drought stress (Nunes et al., 2008) and in transgenic *M. truncatula* lines expressing the trehalose-6-Phosphate Synthase 1 (*AtTPS1*) from *Arabidopsis thaliana* with altered response to water deficit and recovery (Alcântara et al., 2015). Taken together, these observations suggest a profound elastic modification of the cell walls of water stress tolerant cells, perhaps deriving form a modified ratio among cell wall fractions, and should be the object of future studies.

Similar levels of somatic embryogenesis were observed in the PEG and control calli, however, calli in the PEG treatment were distinctly green in color compared with the control. This might be consistent with more robust embryogenic callus in the PEG compared to the control treatments (**Figures 1, 2**), which is not surprising since MANA medium is not conductive to full maturation of the somatic embryos formed in *M. truncatula* (Ochatt et al., 2013; Ochatt and Revilla, 2016). It may also reflect the fact that *M. truncatula* is adapted to semiarid conditions and even under severe drought stress, pigment content is not affected (Biswal, 1997; Nunes et al., 2008). PEG also improved somatic embryogenesis in other species (Attree et al., 1995; Igasaki et al., 2003). Both control and PEG treatments resulted in similar levels of somatic embryogenesis, which was consistent with the similar expression levels of *MtSERK* in the two treatments. Note that *SERK1* is highly expressed during embryo induction and early somatic embryo development in *M. truncatula* (Nolan et al., 2009) and in *Araucaria angustifolia* (Steiner et al., 2012). The possible stimulation of somatic embryogenesis in response to PEG treatment is consistent with other reports showing stress-induction of this process (Karami and Saidi, 2010), which in *M. truncatula* may be linked to increases in ABA (Nolan and Rose, 1998).

**FIGURE 7:**
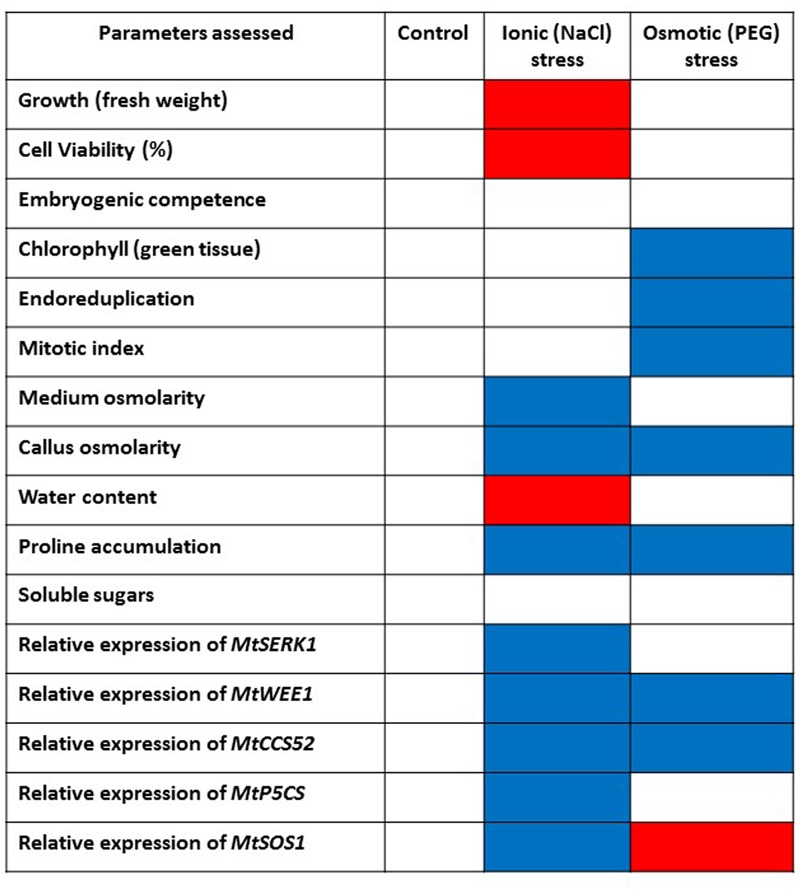
Summarized effects on various parameters of imposing abiotic stress on embryogenic callus of *M. truncatula* following *in vitro* selection. Control callus tissues are compared with NaCl-tolerant callus (Elmaghrabi et al., 2013) and PEG-induced osmotic stress tolerant callus tissues in this study. Blue color indicates increase/upregulation. Red color indicates decrease/downregulation. When non-significant compared to controls white is used.

The physiological, metabolic and gene expression responses of calli to PEG-induced osmotic stress mirrored those found under salt stress treatments (Elmaghrabi et al., 2013) in some respects but not in others, as summarized in **Figure 7**. In contrast to NaCl treatment, PEG treatment did not result in any increase in water content of the calli compared with the control although osmolarity did increase. This could be explained by the differential modes of action between NaCl and PEG, as the high MW of PEG exerts a constant osmotic pressure but does not allow its entry across the wall and hence avoids cell plasmolysis which results in different energy costs and different effects on growth (Munns, 2002). However, soluble sugars did increase suggesting they are a useful marker of both salt and water (osmotic) stress (**Figure 5**). PEG also induced a high level of proline accumulation, which was far higher than the largest proline accumulation under stress induced by NaCl (Elmaghrabi et al., 2013), indicating that this might also be a component of osmotic stress tolerance in *M. truncatula* (**Figure 5B**), as has been found in other species (Deinlein et al., 2014). Validating this hypothesis would require field trials with regenerants from these cultures and goes beyond the scope of this study. However, the expression of *MtP5CS* in callus grown on PEG was similar to the control (**Figure 6D**) and hence does not correlate with increased levels of proline in the PEG treatment. This was surprising given that this gene encodes an enzyme that is central to proline synthesis and that its expression in *M. truncatula* cultures exposed to salt stress was elevated (Elmaghrabi et al., 2013; **Figure 7**). It may suggest that this enzyme is not a key regulatory step in proline biosynthesis under these conditions, or that an initial rise in *MtP5CS* expression early in the culture period was sufficient to elevate proline concentrations and that after 6 months culture, increased gene expression was no longer necessary. In other words, whether modifying proline metabolism and the expression of genes involved in it, such as *P5CS*, may or not be used for engineering drought tolerance, and which approach should be adopted for such modification to be done remains uncertain (Bhaskara et al., 2015).

More predictably, PEG did not induce *MtSOS1* expression which was down-regulated compared with the control. This gene is highly expressed in salt stress conditions as it encodes a protein that functions as a membrane-bound Na^+^ antiporter and contributes to Na^+^ depletion in the cytoplasm (Smith et al., 2010; Feki et al., 2011). Therefore the PEG data indicate a different (non-ionic) pathway leading to osmotic stress tolerance compared with NaCl (ionic) tolerance (**Figure 7**) as reported by Elmaghrabi et al. (2013).

The expression of *MtCCS52* was upregulated by the PEG treatment (**Figure 6E**). In Arabidopsis this gene is a regulator of ploidy level and its expression is positively correlated to endoreduplication. In *M. truncatula* cultures exposed to long-term NaCl treatments this gene was up-regulated (**Figure 7**), alongside an increase in endoreduplication (Elmaghrabi et al., 2013). Given the clear evidence for endoreduplication in the osmotic stress-resistant cultures here (**Figure 3**), the upregulation of *MtCCS52*is consistent with its role in Arabidopsis (Vanstraelen et al., 2009).

That *MtWEE1* expression was more highly expressed in the PEG treatment suggests that this gene may have a role in maintaining normal growth in a treatment that mimics osmotic stress conditions. *WEE1* kinase might be necessary to regulate normal cell size in the face of ion toxicity and osmotic (non-ionic) water stress although this could only be resolved by exposing calli from *M. truncatula wee1* knockouts to these treatments. Alternatively, as a gene that is expressed in the DNA damage and DNA replication checkpoints, it may be induced in response to either single strand or double strand DNA breaks as it is in Arabidopsis (De Schutter et al., 2007). However, Gonzalez et al. (2004) observed high expression of *LeWEE1* in tomato (*Lycopersicon esculentum* Mill.) which was correlated with endoreduplication during fruit development. Our results do not seem to indicate that there has been irreversible DNA damage due to the osmotic stress imposed on callus, and it would therefore be legitimate to link this to *WEE1* expression and its role in replication checkpoint and DNA damage and the possibility that the PEG concentration used and the long-term culture on it resulted in priming (Singh et al., 2015). Other genes could also be involved in the process though, and, in this respect, in order to protect their gene integrity from DNA damage plants are capable of activating a specific response system that regulates the cell cycle, but also DNA repair and programmed cell death where genes such as *Suppressor Of Gamma response 1* (*SOG1*) (Yoshiyama et al., 2014) and *Breast Cancer 1* (*BRCA1*) (Block-Schmidt et al., 2011) are known to play a central role in DNA repair, chromosome segregation and chromatin remodeling So the increase in *MtWEE1* seen here may be both linked to the increase in endoreduplication and required to protect the cells from DNA damage induced by the PEG-induced osmotic stress treatment.

## Conclusion

The data reported in this study of responses to 10% PEG compared with no PEG controls indicates that at this level of osmotic stress it is possible to induce a high level of embryogenesis with no penalty on growth rate. This appears to be achieved by up-regulating protective mechanisms such as the production of osmoprotectant solutes and switching on the expression of *MtWEE1.* The increase in *MtWEE1* and *MtCCS52* expression may cause an increase in endoreduplication while protecting the cells against the potentially damaging effects of the osmotic stress on DNA integrity.

## Author Contributions

HR, DF, and SO designed the project and the experiments. AE performed the experiments. All authors analyzed the data, read and approved the manuscript.

## Conflict of Interest Statement

The authors declare that the research was conducted in the absence of any commercial or financial relationships that could be construed as a potential conflict of interest.
